# Perspectives on a Novel Culturally Tailored Diabetes Self-Management Program for African Americans: A Qualitative Study of Healthcare Professionals and Organizational Leaders

**DOI:** 10.3390/ijerph191912814

**Published:** 2022-10-06

**Authors:** Meng-Jung Wen, Martha Maurer, Luke Schwerer, Nassim Sarkarati, Ugboaku Maryann Egbujor, Jenna Nordin, Sharon D. Williams, Yao Liu, Olayinka O. Shiyanbola

**Affiliations:** 1 School of Pharmacy, University of Wisconsin, Madison, WI 53705, USA; 2 School of Medicine and Public Health, University of Wisconsin, Madison, WI 53705, USA

**Keywords:** African Americans, diabetes, self-management, peer support, stakeholder engagement

## Abstract

Background: There is an urgent need for culturally tailored diabetes self-management education to improve health outcomes in African Americans, especially given the disproportionate prevalence of diabetes and medication non-adherence. Stakeholder engagement can guide and enrich the development of these interventions by integrating content directly addressing barriers to African Americans’ adherence with existing community-based diabetes self-management education programs. The aim of this study is to explore stakeholder perspectives on a novel culturally tailored diabetes self-management program for African Americans. Methods: Thirteen semi-structured individual interviews were conducted in a large Midwestern U.S. city with healthcare professionals and organizational leaders serving African American communities and/or providing diabetes education. Transcripts were analyzed using directed content analysis with the Consolidated Framework for Implementation Research and inductive content analysis. Results: Five overarching themes were identified: (1) fulfill needs among stakeholders, (2) creating a supportive and trusting environment to address distrust, (3) building relationships and empowering peers, (4) logistical organization barriers to program implementation and (5) challenges to program acceptance by participants. Conclusion: Stakeholders delineated how the new culturally tailored diabetes self-management program aligned with the needs of African American patients. Perceived challenges and corresponding strategies to address barriers to participation were identified to inform program implementation and sustainability.

## 1. Introduction

Diabetes is a leading cause of death in the United States, accounting for over 100,000 deaths annually. Diabetes affects 37.3 million Americans and is a condition that continues to rapidly rise in prevalence, requiring complex management to avoid complications that contribute substantially to increased healthcare costs [1,2]. Further, there are major racial and ethnic disparities associated with diabetes morbidity and mortality. The prevalence of type 2 diabetes in non-Hispanic Blacks was 11.5% compared to 7.7% for non-Hispanic whites in 2018. Non-Hispanic Blacks are also more likely to develop diabetic kidney disease and retinopathy, and have the highest rates of hospitalization and mortality related to diabetes [2].

There are stark disparities indicating disproportionately higher medication nonadherence among non-Hispanic Blacks or African Americans (AAs) [3,4,5], which contributes to higher hospitalization rates, greater emergency room visits, and increased mortality rates [6]. Barriers to medication adherence among AAs with diabetes include personal experiences of discrimination in healthcare and distrust for healthcare providers, limited confidence to ask healthcare provider questions about medications, and personal or fatalistic beliefs about the causes of diabetes [7,8,9,10,11]. Hence, there is an urgent need to implement diabetes self-management interventions culturally tailored to AAs [6]. Previous culturally specific educational interventions for AAs have focused on dietary cooking behaviors, the importance of family support, education about symptoms, the effect of stress and depression, and utilization of groups for program delivery [12]. However, very few interventions address medication nonadherence [13], a key factor associated with increased mortality and morbidity from diabetes.

To directly address barriers to medication adherence among AAs with type 2 diabetes, our team developed and implemented a theory-driven novel program called Peers Supporting Health Literacy, Self-Efficacy, Self-Advocacy and Adherence (Peers LEAD) [14,15,16]. It is a culturally tailored program for AAs which provides one-on-one peer support and behavioral skill development to improve medication adherence. For the delivery of program contents, we utilized several principles such as active learning strategies, simple plain language, visualization and small group discussions in the group education sessions with peers to address health literacy barriers. Two pilot studies indicated that Peers LEAD led to a 10% increase in self-reported medication adherence over 3 months, a 30% reduction in negative beliefs about medicines and diabetes, a 23% increase in self-efficacy for medication use, and a 22% increase in social support over 3 months [15,16]. Despite these promising results, a significant limitation of Peers LEAD is that the program focuses primarily on diabetes medication adherence for AAs and does not address other important aspects of diabetes self-management such as diet and lifestyle.

Therefore, we developed a novel culturally tailored diabetes self-management education (DSME) for AAs called Peers’ Experience in Communicating and Engaging in Healthy Living (Peers EXCEL) by combining Peers LEAD with an existing community-based DSME program, Healthy Living with Diabetes (HLWD). The American Diabetes Association strongly endorses diabetes self-management education (DSME) [17], which has been proven to improve health outcomes (i.e., decreases in hemoglobin A1c and fasting blood glucose), quality-of-life, and facilitate lifestyle and behavioral modifications in patients with diabetes [17,18,19]. Yet, DSME programs have not previously effectively addressed the main barriers to medication non-adherence among AAs. We hypothesized that the integration of Peers LEAD and HLWD may bridge the gap in this intervention, creating a culturally tailored, community-based DSME program for AAs.

Our long-term goal is to implement Peers EXCEL and evaluate its impact on medication adherence and glycemic control among AAs. The 2022 National Standards for DSME specify that healthcare professional and organizational perspectives need to be included in developing DSME programs since such input has proven effective in improving health outcomes, increasing patient participation, and promoting the sustainability of DSME programs [20]. Leveraging stakeholders’ recommendations and strategies not only strengthens participation among potential populations but promotes sustainable diabetes self-management programs [21,22,23]. Therefore, our first step was to conduct individual semi-structured interviews with healthcare professionals and community-based organization leaders who provide DSME to explore their perspectives on the novel culturally tailored DSME program for AAs, Peers EXCEL.

## 2. Materials and Methods

### 2.1. Description of the Peers EXCEL Intervention

We developed Peers EXCEL by combining Peers LEAD, a peer supported program for AAs focused on diabetes medication adherence, with Healthy Living with Diabetes (HLWD), a community-based DSME program, which is readily available in a Midwestern state.

In the Peers LEAD program, AAs who have type 2 diabetes and are adherent to their medications (Peer Ambassadors) are paired with AAs with type 2 diabetes who are nonadherent (Peer Buddies). The peer support includes, but is not limited to, initial in-person and phone follow-ups, addressing misperceptions of medicines and diabetes, sharing experiences in managing diabetes and medications, discussions on medication management strategies and proactive support of self-advocacy in patient-provider interactions.

HLWD is an evidence-based program which addresses several aspects of diabetes care including diet, exercise, and medications. This program is 6-weeks long, with workshops hosted for 2.5 h weekly. Although HLWD includes a 20-min activity on medications, it does not effectively address the main barriers related to medication nonadherence among AAs.

**Figure 1:**
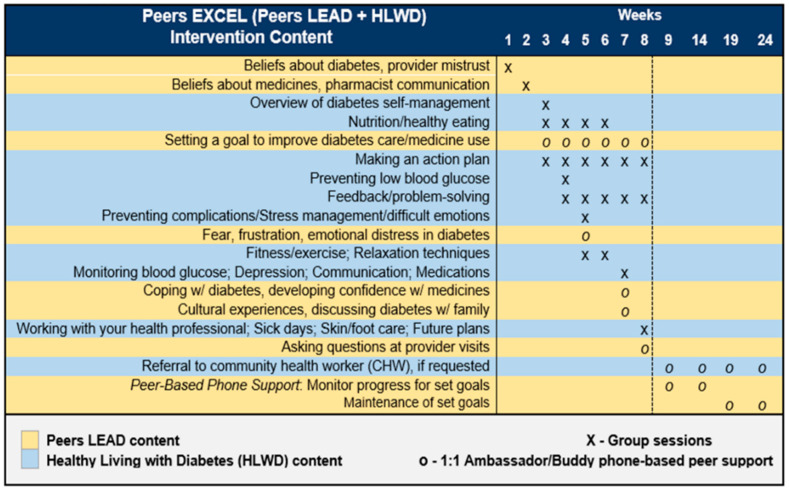
Peers EXCEL (Peers LEAD + HLWD) Intervention Content.

Peers EXCEL is a program that integrates Peers LEAD with HLWD content, by adding two group sessions in weeks 1 and 2 led by a pharmacist and healthcare provider to address African American beliefs and misperceptions about medicines and diabetes. The program is intended for individuals who self-identify as AA who have been diagnosed with type 2 diabetes for more than 1 year and self-reported being prescribed one oral or injectable diabetes medication. As well, the program is intended for buddies who: (1) Self-reported nonadherence on the Adherence to Refills and Medications Scale for Diabetes (ARMS-D) scale and (2) Most recent HbA1c is higher than 7.6% at baseline screening. And for ambassadors, we will include AAs who self-report adherence on the ARMS-D scale. This is followed by 6 weeks of HLWD diabetes self-management content which will be led by two AA HLWD-trained group leaders, in addition to peer support focused on specific topics related to addressing health beliefs, family role in self-management, provider communication about medicines, and maintaining cultural experiences in diabetes self-management. Resources including a program book and referral to community health workers about participants’ needs on social determinants of health will be provided. Figure 1 provides complete details about Peers EXCEL.

### 2.2. Research Design

We used descriptive qualitative methodology to investigate perceptions regarding Peers EXCEL from healthcare providers and organizational leaders who are key stakeholders in implementing the intervention. Individual semi-structured interviews were conducted virtually via a web-based platform in a large Midwestern City from December 2020 to October 2021. Semi-structured interview guides were developed and informed by the Consolidated Framework for Implementation Research (CFIR) to identify strategies that could support and facilitate implementation of Peers EXCEL. Three domains in the CFIR model were selected: (1) intervention characteristics (relative advantage, cost, design, complexity), (2) outer setting (individual needs, barriers), and (3) inner settings (organizational culture, capacity for change, compatibility, readiness) and adapted for the two separate groups of participants based on the domains most relevant to their roles, responsibilities, and tasks in implementing the proposed intervention.

### 2.3. Data Collection

The study was determined by the PI’s University Institutional Review Board to be exempt because it only involved interview procedures. We used snowball sampling, a type of convenience sampling, and collaborated with community partners, including recruitment by word of mouth, to invite healthcare professionals and organizational leaders to participate in the interviews. The eligibility criterion for the healthcare professionals was having had experience treating or serving patients with diabetes, preferably patients from the African American community. The eligibility criteria for organizational leaders were either having had experience hosting HLWD in the past or representing an organization serving the African American community that was identified by the research team as a potential host for Peers EXCEL in the future. Interviewers were research assistants who had been trained by the PI in conducting qualitative interviews, which involved reviewing literature on appropriate interviewing techniques, reviewing the interview guide in detail, and role-playing an interview with the PI to demonstrate mastery of interview skills. Research team members reviewed the purpose, format, and length of the interview to assure participants about confidentiality issues and voluntary participation in the study to obtain verbal informed consent. Each interview lasted up to 60 min and was audio-recorded and transcribed verbatim by a professional transcriptionist. Sample interview questions are provided in Table 1.

### 2.4. Data Analysis

Both directed content analysis using the Consolidated Framework for Implementation Research (CFIR) and inductive content analysis were used to identify facilitators and barriers to program implementation [24]. We sought to focus our analysis on answering two key research questions: (1) What will facilitate the success of implementing Peers EXCEL at the participating site? (2) What challenges may be encountered when implementing Peers EXCEL at the participating site? The CFIR was adopted to guide our analysis to demonstrate key components and provide successful strategies to facilitate program implementation.

Two trained research members used NVivo 10 (QSR International-Melbourne) to analyze the qualitative transcript independently. First, in the process of open coding, research members read the transcript line-by-line to develop a codebook with codes and themes. Second, axial coding was used to compare existing themes with new themes and add the new concepts until data saturation [25]. Last, peer debriefing was conducted regularly to ensure the consistency and the interpretation of themes and codes derived from data. To ensure all themes and codes captured participants’ experiences, we held an event to disseminate the results of the analysis to achieve trustworthiness by conducting member checking [26].

## 3. Results

### 3.1. Brief Description of Participants

A total of 13 interviews were conducted with seven healthcare professionals and six organizational leaders (Table 2). Seven healthcare professionals including pharmacists, dieticians, diabetes educators, and chief medical officers who serve AA patients in practice were included to provide the feedback on the value of Peers EXCEL. The organizational leader stakeholders served in medical clinics, community health centers, pharmacies, and health services all within a large city in the Midwest. Many of the stakeholders worked with the AA community in addition to non-AA communities. Many also had experience with diabetes education. To gather diverse perspectives on implementing Peers EXCEL, we conducted four interviews with sites who had previously implemented HLWD (past host sites (PHS)), and two interviews with possible future host sites (FHS) of Peers EXCEL.

### 3.2. Key Stakeholders’ Perspectives of Peers EXCEL

Our analysis identified five major themes related to the implementation of Peers EXCEL: (1) fulfill needs among stakeholders (2) creating a supportive and trusting environment to address distrust, (3) building relationships and empowering peers (4) logistical organization barriers to program implementation and (5) challenges to program acceptance by participants. Table 3 portrays themes, subthemes and representative quotes.

#### 3.2.1. Fulfill Current Needs among Stakeholders

The advantages of Peers EXCEL perceived by stakeholders well-aligned with the missions of their organizations. Capacity to provide diabetes education to anyone despite barriers to address health inequity is recognized as a shared goal for both healthcare and local organizations.

*“[HLWD] is really open to anyone in our community. So, we don’t limit it to just those who get care at [this specific health system]. I think it’s really important to get them that education if they can’t afford it, they don’t have access.”—PHS 4*

Peers EXCEL also fills the existing gaps that organizations were lacking. The advantages of utilizing existing partnerships and leveraging new partnerships with healthcare and local organizations were recurring themes elicited from both healthcare professionals and organizational leaders. Healthcare professionals recognized that partnering with a program like Peers EXCEL would complement clinical care and fill important knowledge gaps resulting from cultural barriers in diabetes self-management topics like diet and exercise. Healthcare professionals generally perceived limited time and fragmented care are barriers to achieving comprehensive care. Additional programs like Peers EXCEL not only dives into long-term lifestyle changes but take advantage of a team-based care approach to ensure holistic care for patients with diabetes.

Sites with experience leading HLWD shared many advantages for future implementation of Peers EXCEL. These advantages included having a location and availability, supporting training, having the resources to provide food and transportation for program meetings using HLWD infrastructures. In addition, partnering with other community organizations not only add flexibilities to support the program but help overcome barriers to participant access to DSME.

*“Our location was centrally located in the community. We had an open-door policy with our residents. We were able to provide all the ingredients of a successful workshop…So definitely making sure that we offer multiple times for participants is one of the things and making sure that a snack or a meal of some sort is provided is the other…coordinating transportation is not necessarily the primary focus.”—PHS 1*

#### 3.2.2. Creating a Supportive and Trusting Environment to Address Distrust

The importance of peer support was emphasized for creating a space in which participants could feel comfortable having conversations about their health. Stakeholders perceived a strong benefit from Peers EXCEL in facilitating trust and creating a safe space for participants. Stakeholders also believed that listening to and learning from peers would help patients relate to each other’s experiences, which would add additional value to the knowledge provided from healthcare professionals. Peer support was also described as an opportunity for patients to voice their concerns to others who care and who have first-hand knowledge of possible solutions to address challenges.

*“With the peer support, they can get into more specific issues if they are really having troubles with maintaining their diet or maintaining their medications… they would have someone that they can voice their concerns to plus someone who would be there that can give them pointers on how to get around it. Just having the classes alone, you might get that, but a more one-on-one would, give that extra level of support.”—HCP 6 (Pharmacist)*

In addition to supporting participants with a safe place to talk about concerns, stakeholders also valued that Peers EXCEL provides additional opportunities and more time to engage with healthcare professionals about disease-related content. Topics such as diabetes perceptions, myths about medicines, and diabetes management ideas were perceived as helpful discussions which may not be covered in-depth by current diabetes self-management education class contents for AAs.

*“I do think that [Peers LEAD] was able to let the participant have more of a voice, exploring their perceptions and their ideas related to how they handle their diabetes. I think that is very, very good …. I know the other program has more structured sessions, which sometimes the timeline gets to be a little bit short for really letting people really understand those areas. So I think that maybe having that peer support in between could help reinforce some of those concepts that might not be able to be put in the actual class sessions….”—HCP 2 (Nutritionist)*

#### 3.2.3. Building Relationships and Empowering Peers

Healthcare professionals perceived the idea of utilizing peer support seems to help participants connect and motivate each other. In terms of peer discussion, the shared experiences of eating style within communities could be incorporated into program contents and provides options or opinions to address it. Along with the shared benefits of peer support, sites with experience leading HLWD perceived Peers EXCEL as an opportunity for patients to build relationships with their peers and reduce social isolation among AAs living with diabetes.

*“In HLWD, they share their struggles, they really feel connected to others in the class. They’re not in this battle alone. They have others they can reach out to that know what it’s like to live with diabetes”—PHS 4*

Stakeholders described that patients in Peer EXCEL may increase their level of engagement with their diabetes management as a result of connecting with peers who can provide social and emotional support, and enhance motivation to improve their health, especially in building diabetes self-management skills.

*“I think it’s going to help a lot with self-management, and eventually that gets reflected into an A1c... Really, it’s about adapting a healthier lifestyle, which is more important than just an A1c., it’s going to help with patient engagement. And that is one of the first steps in improving diabetes care.”—HCP 7 (Provider)*

*“We recognize it’s important that individuals that want to have better management of their lives learn from their peers and individuals that can resonate with their story.”—PHS 1*

#### 3.2.4. Logistical Organization Barriers to Program Implementation

Several concerns were shared about a lack of organizational resources or capacity to successfully support a program like Peers EXCEL. In particular, important tasks such as patient recruitment, session set-up, and food coordination would require more staff than they currently have available.

*“The position of the coordinator role is not a state-mandated service at this time, and so it’s unlikely that more resources will be allocated to that position, and there is no capacity for that coordinator to increase time and do more programs than what we already have.”—PHS-3*

Stakeholders also shared concerns about scheduling challenges their organization would face in providing sufficient flexibility and timing of meetings that would allow Peers EXCEL participants to commit to attend the entire length of the 8-week program.

*“...in general, it’s time, work schedule. You know, some people are working the second shift or third shift. These are the barriers that they may not participate in the program.”—HCP 6 (Pharmacist)*

#### 3.2.5. Challenges to Program Acceptance by Participants

Additional logistical barriers were noted for participants, in addition to recruitment difficulties stemming from a lack of participant trust, lack of awareness of programs and relatively low numbers of AAs living in parts of the Midwest.

*“It would be really nice if we could reach out to our African American community in [a city in Midwest], our surrounding areas, and ask them what they’re comfortable with… Are you comfortable coming in to take a class inside the healthcare setting, or would you rather have it within your community?”—PHS 4*

Organizational leaders demonstrated the importance of promoting affordability of participating in the program. To enhance commitment to continued participation, leaders also proposed the ideas of adding a small monetary fee. Some logistical components that were considered important for participants included providing a snack or meal during the meetings, in addition to offering transportation to attend the program sessions.

*“...a lot of my patients do have difficulty getting transportation. So I think that might be a barrier.”—HCP 3 (Pharmacist)*

Another important barrier to program participation by AAs included a lack of trust in those leading the Peers EXCEL program, particularly when implemented in communities without a large AA population. Furthermore, they discussed that it can be difficult to attract adequate numbers of participants in those communities and would require additional outreach efforts to increase awareness of the program in AA communities. Stakeholders mentioned this could be possible through program ambassadors, healthcare providers, and diabetes educators, in addition to identifying spaces where AAs would prefer to gather to participate in the program.

*“[Patients] may be more willing to come if they really trust the people that’s involved in it. So, who’s leading the program, how the outreach is, those can either enhance or can be barriers to participation. But if someone that you know and trust asks you to do it, you’re more apt to do it.”—HCP 2 (Nutritionist)*

*“It would be really nice if we could reach out to our African American community in (a Mid- Western County), our surrounding areas, and ask them what they’re comfortable with… Are you comfortable coming in to take a class inside the healthcare setting, or would you rather have it within your community?”—PHS 44. Discussion*

Our study explored perspectives from key stakeholders regarding implementation of a novel culturally tailored diabetes self-management program for the AA community, Peers EXCEL, using the CFIR framework. When focusing on the CFIR domains of intervention characteristics (relative advantage, cost, design, complexity), (2) outer setting (individual needs, barriers), and (3) inner settings (organizational culture, capacity for change, compatibility, readiness), we identified five major themes, including (1) partnering with local organizations, (2) creating a supportive and trusting environment, (3) building relationships and empowering peers (4) logistical barriers to program implementation and (5) challenges to program acceptance. We plan to use these themes to help pilot the feasibility and acceptability of Peers EXCEL and inform successful Peers EXCEL program implementation and sustainability.

To successfully translate evidence into practice, we used stakeholder engagement to provide input on the Peers EXCEL program. Stakeholder engagement, including experts in clinical, organizational, and behavioral diabetes care, has been recognized as a standard in developing programs to ensure effective DSME program delivery [27]. Consistent with the literature, stakeholders believed that the design of the new intervention (Peers EXCEL), which combines the culturally tailored peer support program (Peers LEAD) with the existing DSME infrastructure from HLWD, is a major strength and could help fill gaps in the fragmented nature of diabetes care within the US healthcare systems [20,22,23]. Seeking input from organizational leaders who had experience in disseminating DSME in community-based settings provided firsthand knowledge of important considerations for Peers EXCEL implementation, in addition to practical strategies to address logistical barriers to promote the feasibility and future sustainability of the program [28,29].

Stakeholders also reported the perceived benefits of having peer support as being complementary to the care provided by healthcare professionals. While healthcare professionals may provide the clinical expertise to properly address diabetes management, especially around the initial diagnosis, there is substantial evidence that peer support plays a valuable role in long term diabetes management since most healthcare providers are not equipped with the time or skills to provide comprehensive behavioral and psychosocial support [30]. In our study, many healthcare professionals described a substantial lack of time and resources to adequately discuss certain important aspects of diabetes management such as diet. Peer support provides a feasible, lower cost, and effective method for supporting long-term diabetes management through shared goal setting and problem solving using culturally tailored strategies. A 2017 systematic review did not find healthcare professional education to be superior to peer educator interventions [31]. This study suggests that combining both approaches in diabetes management, especially among marginalized populations, may be more effective than either alone. Furthermore, stakeholders suggested that peer support may increase participants’ comfort level in approaching their doctors with questions about their health and develop more collaborative relationships with healthcare providers.

Our findings also highlighted the value that stakeholders placed on Peers EXCEL as a resource to provide culturally tailored content for AA patients to empower themselves in diabetes self-management. Peers EXCEL program may help reduce the barriers AA communities face in accessing diabetes self-management information, building connections with peers, as well as addressing mistrust about the healthcare system. This finding is consistent with prior studies [9,15,32,33,34], which indicate that addressing healthcare provider mistrust is an important intervention component in culturally tailored interventions among AAs to enhance disease self-management and medication adherence, and further reduce diabetes disparities.

Despite the many perceived benefits of Peers EXCEL, stakeholders also expressed concern regarding several logistical challenges for implementation. For example, recruiting AAs to participate in the program could be difficult due to stakeholders’ perceived differences in education, fear of the unknown, less access to care [35] and lack of program awareness. It is possible that there are fewer AAs available to recruit for the Peers EXCEL program due to barriers that prevent AAs from accessing diabetes care programs, which could refer them into Peers EXCEL. Additionally, differences in available resources at Peers EXCEL host sites present unique challenges to program implementation. For instance, several past host sites highlighted that they lacked personnel to run the program. Furthermore, these sites indicated that providing healthy foods during program sessions required additional time and resources to coordinate. These barriers are consistent with findings from the 2006 California Health Care Foundation Report, *Building Peer Support Programs to Manage Chronic Disease: Seven Models for Success*, which noted various costs associated with peer-based support programs like Peers EXCEL. Though community stakeholders highlighted several anticipated challenges with program’s implementation, they also described possible strategies for overcoming these barriers, including building connections with healthcare providers and AA community to increase awareness [36].

There were some limitations of our study. Participants were recruited through convenience and snowball sampling in one Midwestern state in the U.S. However, our participants were in communities with or without African Americans as the majority racial and ethnic group. Findings from this study could counterbalance the prospective issues of recruitment for future program implementation. To enhance the diversity of our responses, we purposefully sampled organizational leaders with prior experience in leading the HLWD DSME program including those without such prior experience, but with whom we plan future collaborations to lead Peers EXCEL. However, future studies may include broadening to include stakeholder perspectives across additional healthcare systems, in addition to AA patients’ prior to implementation to ensure that interventions are aligned with the needs of the community. While many of the stakeholders did not personally identify as AA, all had extensive experiences serving AA patients in their practice or organization and had valuable perspectives to guide our future implementation of Peers EXCEL within the context of their organizations. This study also implicates that more culturally tailored contents of long-term lifestyle change are needed in the current healthcare systems to achieve a comprehensive care for underserved/marginalized communities.

## 4. Conclusions

We identified major themes using key stakeholders’ perspectives related to facilitators, barriers, and strategies to be used in the implementation of a culturally tailored diabetes self-management program for AAs. Healthcare professionals and organizational leaders believed that the Peers EXCEL program would complement clinical care and could contribute to reducing health disparities by providing peer support in address barriers to diabetes medication adherence, diet, and lifestyle management. Peer support was viewed as having the ability to create a safe, open environment to facilitate trust-building and improve diabetes self-management among AAs. Potential strategies were identified to overcome logistical barriers to participation and enhance recruitment. Findings from this qualitative study will inform the pilot feasibility and acceptability of implementation of this culturally tailored diabetes self-management program for AAs living with diabetes.

## Figures and Tables

**Table 1:** Interview Questions for Healthcare Professionals and Organizational Leaders.

Healthcare Professionals	
Domain: Meeting the needs of the African American patient population with type 2 diabetes	
	How well do you think Peers EXCEL implemented in the community would meet the needs of African Americans with diabetes in your organization?
	Case: Imagine you are working with an African American patient who had been diagnosed with diabetes for over 5 years and was struggling to bring down their A1C. What challenges do you see patients like this facing? What are they lacking?
	How do you think African Americans served by your organization will respond to your Peers EXCEL?
	What barriers will African Americans with diabetes served by your organization face to participating in the Peers EXCEL program?
Domain: Identifying organizational factors that might influence Peers EXCEL implementation and effectiveness	
	Tell me about what your organization currently offers related to diabetes self-management services for patients. (Adapted to align with each type of HCP)
	How is Peers EXCEL similar or different than other similar existing programs in your setting?
	How does Peers EXCEL align with your organization’s goals?
	Would you be willing to refer African American patients with diabetes who are having challenges with taking medicines to a program, such as Peers EXCEL in the community?
	Is there anything you feel is missing from Peer EXCEL that would make it more effective?
**Organizational Leaders**	
Domain: Organization’s experience with hosting HLWD (past host sites)	
	Tell me about your organization’s experience with hosting HLWD
	Tell me how/why your organization first become involved with hosting HLWD.
	Tell me what made it convenient for your clientele or community to attend the HLWD sessions.
	Tell me about the barriers that people faced in attending HLWD sessions.
Domain: Organizational goals or activities related to health & diabetes	
	Is Peers EXCEL consistent with your organization’s goals? (both)
	Tell me what your organization currently has in terms of health programs or ministries. (Future host sites)
	Tell me about what makes it convenient for your clientele/community to attend these programs/meetings. (Future host sites)
	Tell me about any barriers that people face to attending these programs/meetings. (Future host sites)
	In what way could Peers EXCEL be useful to the population your organization serves? (both)
	How do you think African Americans with diabetes served by your organization would respond to Peers EXCEL? (Future host sites)
	What do you see as the gaps in diabetes self-management for African Americans in your community? (Future host sites)
Domain: Interest in hosting Peers EXCEL (both)	
	Do you think your organization would be interested in hosting Peers EXCEL in the future?
	What would it take for your organization to be interested in this idea?
Domain: Identifying logistical factors that might influence hosting the new program at the organization (both)	
	What would your organization need to make hosting Peers EXCEL work?
	How would your organization offer/tailor Peers EXCEL to work in your setting?
	Tell me about the barriers or challenges you think there might be for your organization to host Peers EXCEL.
	What barriers would African Americans with diabetes served by your organization face to participating in Peers EXCEL?

Abbreviations: Peers EXCEL—Peers’ Experience in Communicating and Engaging in Healthy Living, HLWD—Healthy Living with Diabetes.

**Table 2:** Stakeholders’ Characteristics (*n* = 13).

Characteristic	
Healthcare Professionals	*n* = 7
Pharmacist	4
Provider	1
Nutritionist	1
Diabetes Educator	1
Organizational Leaders	*n* = 6
Past Host Site	
Health System	2
Non-profit Organization	1
County Agency	1
Future Host Site	
Church	1
Senior Housing Complex	1
Type	
Black-serving Organization	4
Not Black-serving Organization	2

**Table 3:** Stakeholders’ Perceptions of Peers EXCEL.

Themes	Subthemes and Quotes
Fulfill needs among stakeholders	*Program aligns with organizational goals and mission* “Well, my particular organization’s goal is to reach as many people as possible to help them live long and healthy and productive lives. And this program feeds right into it because of... my commitment to diabetes from the standpoint that many African Americans don’t get the same type of education around health and health literacy. And this program would definitely go a long way to meeting that goal…”—HCP 2 (Nutritionist) “My biggest concern is that they’re [rationing diabetes] medicines, which is shortening the supply of the person [from whom they are borrowing medication] …I bring somebody in to talk about the medication, like what happens when you do that, what happens to your body because you’re not getting the full amount that you need so that you could be sustained….They’ll sit and take the time to answer any questions that they have about their medications or about health conditions... So, you can’t beat that, the pharmacist coming to you.”—FHS 2
	*Low capacity of health system increases the need for and relevance of the program* “In a clinical setting, as a provider, we have very limited time to provide education on diet and exercise and all of that counseling that comes along with it that oftentimes we have to ask colleagues to help like diabetic educators, nutritionists. And that can lead to more of a broken treatment or fragmented, so it can leave patients feeling very frustrated.”—HCP 7 (Provider) “Having an additional program complement [HCP education], with bringing in details about culture, cultural experiences, because unfortunately, I’m not in that culture. I did not grow up African American, so I don’t fully understand it. I think it’s a complement to really help with the management and help them see a side that I would not be able to necessarily present to them.”—HCP 5 (Pharmacist)
	*Strengths from Leveraging HLWD Program Infrastructure* “We have a partnership with Wisconsin Institute for Healthy Aging, which is the state license holder [for HLWD] …we thought it would be a good option to expand, and there are a couple of extra tools that are specific to diabetes. So, we saw the need because of the clientele we were already serving, and it was well received.”—PHS 3 “We’ve also partnered with a food co-op. We’ve held [meetings] in a food co-op learning room, which has been really beneficial because then you have the groceries, then [participants] can seek out some healthy snacks.”—PHS 4
Creating a supportive and trusting environment to address distrust	*Helps further facilitate trust and provides a safe space for participants* “When you have a program that is primarily developed around interacting people of color with people of color, that breaks down some of their barriers, and people are able to have a safe space for telling their truth… also [the] trust factor would be so much better. … [Peers] LEAD I feel is way more sensitive to African Americans’ feelings and, the idea that they really can share, more open to sharing themselves.”—HCP 2 (Nutritionist) “There are a lot of literacy issues, and people have problems understanding the machines that they use or how to do it and questions about their insulin or their medications. And they don’t always understand what the doctors are saying, so they need reinforcement to help them understand what the doctor wants them to do.”—FHS 1
	*Provide additional opportunities to discuss disease-related topics with HCPs* “And the more we can educate patients because sometimes the first time they hear it, it’s not motivating. Or they’re in denial yet about having their diabetes. the more that we can hit home and focus on those education pieces, the better. I think this combination would work really well and be very successful for the community of patients that I’ve personally seen. …And I thought it [Peers LEAD] was a really great opportunity to get to know, and maybe break down some barriers or some myths that are within the African American community about medications.”—HCP 3 (Pharmacist) “They want to know about these different things (content of Peers EXCEL) so maybe this is something that will be better for them. They can go back and tell their doctors… They will listen, and they’re definitely going to ask questions. I think that they would enjoy it.”—FHS 2
Building relationships and empowering peers	*Connection and motivation embedded into the program* “I think [participants] will respond well, because it’s not one-sided... how it’s presented to them is that we’re in this together having all the pieces that go together will have someone feeling like they are connected. And they feel like there’s something out there that’s going to help them, because they want to make their lives better... The compassion of the program makes a huge difference, rather than just being a program that has written things, there is an emotional connection to it. that makes a difference.” HCP 1 (Diabetes Educator) “In my experience, African Americans have such a [bias] around eating and family, and their culture is based in that, a lot of them struggle with making healthy decisions and how to adapt a healthier eating style compared to previous. So many of my patient’s report, I want my fried chicken. That’s just what I have to eat. I want my collard greens. I want my corn bread. So I feel really incorporating that, and then potentially having a peer group where they can talk about that, how they address it and what options and opinions they can use for support would be very beneficial.”—HCP 5 (Pharmacist) “it’s kind of like you’re walking around with someone who can relate to you, so you don’t feel like you’re by yourself. And I think that is the biggest advantage of this program, is to have something that is peer led….”—HCP 1 (Diabetes Educator) “I was highlighting that one of the things that we really appreciate or the concept for integrating Peers LEAD with the self-management diabetes workshop is because part of that work is to have those regular engagements with participants…The Peers leading Peers will provide support in following up with those individuals and building that rapport along with the leader so that is more manageable.”—PHS 1 *Provides peer support that reinforces self-management advice from healthcare providers* “The biggest [benefit] with the combined program would be having the Peer Ambassadors, the peer supports. that is different because many of the programs, people come, they hear a speaker, and they may be able to talk to other people, but then they go home. They don’t have any follow-up., that peer support, that person that they can actually ask additional questions to and have a conversation, I think that makes it much different. It can really build trust and trust that the individuals working with them understand their particular situation. …having that peer support person who understands, what people go through, is a very important thing.”—HCP 2 (Nutritionist) “I think sometimes it’s easy for patients to just see healthcare providers as someone who’s like telling you what to do. And I think having that [peer] mentor, is going to be huge to help patients to get motivated and, and truly see the importance of taking care of themselves…having someone in the community that they can relate to, reiterate those same things, would have an impact and maybe it would hit home a little bit more than just a provider telling them that.”—HCP 3 (Pharmacist) “With the peer support, they can get into more specific issues if they are really having troubles with maintaining their diet or maintaining their medications… they would have someone that they can voice their concerns to, plus someone who would be there that can give them pointers on how to get around it… more one-on-one would, give that extra level of support.”—HCP 6 (Pharmacist)
Logistical organization barriers to program implementation	*Lack of capacity and resources for program* “For us to facilitate the workshop as an agency, is much more taxing than to have the community facilitate the workshops... For our community-based organizations that host, they usually already have a population to be served. So it minimizes the work needed around recruitment and promoting. They facilitate that or help to support the facilitation of that.”—PHS 1 “We would need a dedicated staff to do this work… if you’re doing recruitment, set-up and coordinating food. You’re making sure that you can have that guidance…. It can be a lot of heavy lifting.”—PHS 1 “It would be getting other people trained to host the program… We just don’t have a staff for that. It’s very time consuming and we need the staff. And right now, everything is with COVID”—PHS 2 “We recognize that sometimes when we’re talking about healthy eating, individuals don’t have access to healthy items. And so, making sure that we can coordinate that [with a partner organization] on behalf of participants is something else that we think is a huge opportunity when facilitating a workshop but requires a lot of time”—PHS 1
Challenges to program acceptance by participants	*Perceived barriers in recruiting participants* “People have fear about diseases and what they’re going to find or what the doctor is going to do or not do. Put you on a medication, it’s always fear of the unknown”—PHS 2 “I think with any population, you know, access to care. If they’re not familiar or comfortable with the healthcare setting. Maybe they’re not getting the care that they need right now”—PHS 4 “I think the biggest barrier is really people knowing about the program. We really do a wide variety of outreach strategies, but still, I think not everybody knows of our programs, and that’s always a factor. We want people to hear about it…”—PHS 3 *Making participation convenient* “Making sure that we offer multiple times for participants is one of the things and making sure that a snack or a meal of some sort is provided is the other. But coordinating transportation is not necessarily the primary focus. Although when needed, it was something that we helped to provide support on.”—PHS 1 “They’re free. So there isn’t that cost barrier…. churches and that sort of thing or other sites that would want to host it”—PHS 1 “The participants pay $10 (for the program) …It used to be free. I opened it up free, but then I’d have everyone sign up…the first day I would have like four people. This is too easy not to show up. If you put some monetary value to it, I see there’s a little better commitment.”—PHS 2 *Increasing community awareness of the program* “Having it being known and having that success behind you and having some program ambassadors that have taken this program as great word of mouth that, you know, this is great. You should do it too, you know, that kind of thing.”—PHS 3 “We sometimes call the physician. But again, they have to be reminded to tell their diabetic patients that we offer this class. And I work with the diabetic educators as well, telling them we have this class. So we try to cross as many paths as possible to get people involved to take it”–PHS-2

## Data Availability

The data for the study are available from the corresponding author on reasonable request.

## Abbreviations

Hemoglobin A1c—A1C; PHS—Past host site; FHS—Future host site.
